# Supplementary material for: Large-scale replication study reveals a limit on probabilistic prediction in language comprehension
Source: eLife. 2018 Apr 3;7:e33468. doi: 10.7554/eLife.33468 (PMC5896878; doi:10.7554/eLife.33468)
Supplement: Supplementary file1. — Supplementary Table 1 contains the sentence materials with cloze probabilities (0-100%) of articles and nouns, along with post-noun sentence endings, comprehension questions and expected answer. Of note, because expectedness of the noun is here determined by the cloze value of the preceding article, there are three items (28, 29 and 49) in which the unexpected noun has a cloze that is equal to or higher than the expected noun. This has no repercussions for the statistical results because the noun-analysis was based on noun cloze. Supplementary Table 2 contains the List of changes to the materials used by Delong et al. (2005). Supplementary Table 3 contains detailed information about participants, trial numbers and EEG recording equipment per laboratory. [file elife-33468-supp1.docx]

| *Supplementary Table 1.* Sentence materials with cloze probabilities (0-100%) of articles and nouns, along with post-noun sentence endings, comprehension questions and expected answer. N.B.: Because expectedness of the noun is here determined by the cloze value of the preceding article, there are three items (28, 29 and 49) in which the unexpected noun has a cloze that is equal to or higher than the expected noun. This has no repercussions for the statistical results because the noun-analysis was based on noun cloze. | | | | | | | | | | | | |
| --- | --- | --- | --- | --- | --- | --- | --- | --- | --- | --- | --- | --- |
| Item | Sentence context | Relatively expected | | Relatively unexpected | | Relatively expected | | Relatively unexpected | | Sentence Ending | Question | Intended answer |
|  |  | article | cloze | article | cloze | noun | cloze | noun | cloze |  |  | 1-Y, 2-N  (but see Table 2) |
| 1 | The old wives' tale says that if you want to keep the doctor away then you should eat | an | 82 | a | 0 | apple | 97 | carrot | 0 | a day. |  |  |
| 2 | For the snowman's eyes the children used two pieces of coal. and for its nose they used | a | 98 | an | 0 | carrot | 100 | apple | 3 | from the fridge. | Did they build a snowman? | 1 |
| 3 | It was difficult to understand the foreign professor because he had | an | 55 | a | 39 | accent | 100 | lisp | 7 | when he spoke. |  |  |
| 4 | Katie did not like to say words with the letter s because she spoke with | a | 86 | an | 0 | lisp | 93 | accent | 20 | and was embarrassed. |  |  |
| 5 | Dale was very sorry for what he had said to Bernadette and he knew that he owed her | an | 89 | a | 0 | apology | 93 | cheque | 0 | because she was still upset. | Was Dale sorry? | 1 |
| 6 | The bakery did not accept credit cards so Peter would have to write | a | 93 | an | 0 | cheque | 100 | apology | 0 | to the owner. |  |  |
| 7 | Although the idea of flight was as old as the hills. the Wright Brothers were the first people to build | an | 77 | a | 18 | aeroplane | 80 | kite | 0 | that actually flew. |  |  |
| 8 | The day was breezy so the boy went outside to fly | a | 61 | an | 0 | kite | 97 | aeroplane | 34 | in the park. | Was the day breezy? | 1 |
| 9 | Surrounded by mountains. Lola shouted across the valley and heard | an | 75 | a | 2 | echo | 93 | bird | 3 | in the distance. |  |  |
| 10 | The tweeting in the treetops sounded like | a | 25 | an | 0 | bird | 80 | echo | 3 | to Melissa. |  |  |
| 11 | When the scuba diver saw the tentacle. he quickly realized that the creature under the rock was | an | 84 | a | 9 | octopus | 87 | fish | 7 | in hiding. | Did the diver see a treasure? | 2 |
| 12 | Marge hated baiting the hook. but she knew that it was the only way to catch | a | 66 | an | 2 | fish | 100 | octopus | 13 | without using a lure. |  |  |
| 13 | On a street corner in Paris Kim had her portrait painted by | an | 61 | a | 36 | artist | 97 | student | 0 | for three euros. |  |  |
| 14 | Betsy had no desire to enter the working world, so decided to stay at the university and remain | a | 73 | an | 5 | student | 90 | artist | 0 | for a few more semesters. | Did Betsy find a job? | 2 |
| 15 | Her grandfather always stressed how important it was for Sophia to go to school. because he had never had the opportunity to receive | an | 59 | a | 27 | education | 90 | book | 0 | while growing up. |  |  |
| 16 | James is an avid reader. so for his birthday his sister decided to give him | a | 75 | an | 2 | book | 93 | education | 0 | about Africa. |  |  |
| 17 | When the representative retired in the middle of his term. the state was forced to hold | an | 70 | a | 20 | election | 62 | parade | 0 | in his district. |  |  |
| 18 | Without the floats and the marching bands it wasn't much of | a | 95 | an | 0 | parade | 87 | election | 0 | this year. |  |  |
| 19 | When her husband started staying late at the office every night, Joanne began to suspect that he was having | an | 98 | a | 2 | affair | 100 | fight | 0 | with someone at work. |  |  |
| 20 | Whenever Josh had too much to drink. he became belligerent and would try to start | a | 75 | an | 9 | fight | 90 | affair | 0 | with someone in the bar. | Would Josh sing if drunk? | 2 |
| 21 | Instead of giving her money whenever she asked for it. Rachel's father thought that it would be a real lesson in economics for her if every week she received | an | 60 | a | 9 | allowance | 77 | pound | 3 | to spend as she wished. |  |  |
| 22 | Andrew complained that the only place he could afford was Poundland, after his grandmother gave him | a | 52 | an | 2 | pound | 53 | allowance | 17 | for helping her. |  |  |
| 23 | When she was filling out the paperwork, Merrill had to write down the name of someone who could be contacted in case of | an | 70 | a | 0 | emergency | 93 | fire | 3 | while she was at work. |  |  |
| 24 | Harris saw smoke billowing out of his neighbours' home and immediately got on the phone to report | a | 75 | an | 0 | fire | 93 | emergency | 40 | at their address. | Did Harris see smoke? | 1 |
| 25 | Charlie's wife told him that she was tired of pressing his shirts and that he would have to learn how to use | an | 59 | a | 2 | iron | 97 | hanger | 0 | one of these days. |  |  |
| 26 | I wanted to put my coat in the closet but I could not find | a | 84 | an | 0 | hanger | 87 | iron | 0 | anywhere. |  |  |
| 27 | No matter how safely you drive your car chances are someday you'll be involved in | an | 70 | a | 27 | accident | 97 | race | 0 | with another vehicle. |  |  |
| 28 | When we saw the finish line and the pylons sectioning off part of the road we knew that there must have been | a | 61 | an | 36 | race | 27 | accident | 67 | earlier that day. |  |  |
| 29 | Margot had finally decided to buy a house in the suburbs after a year of renting | a | 66 | an | 11 | flat | 90 | apartment | 90 | in the city. | Did Margot want to buy a car? | 2 |
| 30 | Instead of recording a live album the band decided they would have more control if they recorded in | a | 77 | an | 0 | studio | 90 | apartment | 0 | without a lot of background noise. |  |  |
| 31 | Sue had wanted to go to Tim's birthday party but she was still waiting for | an | 73 | a | 11 | invitation | 77 | message | 0 | from him. |  |  |
| 32 | When I called his house Nolan was not home but his mother said she could relay | a | 64 | an | 0 | message | 80 | invitation | 3 | to him. |  |  |
| 33 | The highlight of Jack's trip to India was when he got to ride | an | 73 | a | 11 | elephant | 53 | bicycle | 0 | in the parade. |  |  |
| 34 | You never forget how to ride | a | 95 | an | 0 | bicycle | 97 | elephant | 30 | once you've learned. |  |  |
| 35 | Because it frequently rains in London it's a good idea to always carry | an | 93 | a | 5 | umbrella | 97 | newspaper | 0 | with you. |  |  |
| 36 | As he walked past the corner drugstore on his way to work Carl skimmed the headlines and decided to go ahead and buy | a | 75 | an | 2 | newspaper | 77 | umbrella | 7 | when he saw what the forecast was. | Was Carl going to work? | 1 |
| 37 | Frank wanted to design a very modern house so he sought advice from | an | 82 | a | 11 | architect | 80 | plumber | 0 | to select the fixtures. |  |  |
| 38 | When the pipe broke in the bathroom Felicia looked through the phonebook to find | a | 93 | an | 0 | plumber | 93 | architect | 0 | who could come and take a look at the job. |  |  |
| 39 | Because Bart did not clean his wound properly he ended up getting | an | 93 | a | 2 | infection | 97 | tattoo | 0 | on his leg. |  |  |
| 40 | The Hell's Angel rolled up the sleeve of his leather jacket to show the girls where he had gotten | a | 55 | an | 0 | tattoo | 93 | infection | 0 | the previous night. |  |  |
| 41 | In order to get into an R-rated movie children under 17 must be accompanied by | an | 91 | a | 9 | adult | 100 | parent | 7 | or legal guardian. |  |  |
| 42 | Older children often have a harder time than younger children dealing with the loss of | a | 91 | an | 0 | parent | 37 | adult | 13 | who has cared for them. |  |  |
| 43 | Jeffrey mailed the letter without | a | 86 | an | 2 | stamp | 93 | envelope | 40 | so the post office would never deliver it. | Did Hannah mail the letter? | 2 |
| 44 | When Wendy went to pay for the birthday card the clerk pointed out that she had forgotten to pick up | an | 77 | a | 0 | envelope | 90 | stamp | 47 | along with it. |  |  |
| 45 | The professional photographer was disqualified from the photo contest because the rules explicitly state that you must be | an | 73 | a | 5 | amateur | 67 | beginner | 17 | in order to compete. |  |  |
| 46 | There are always ways to improve your game whether you're an advanced tennis player or merely | a | 82 | an | 16 | beginner | 63 | amateur | 20 | you just need to practice a lot. |  |  |
| 47 | Amelia did not want to go to the bar with her friends after seeing the movie so she made up | an | 91 | a | 7 | excuse | 100 | story | 13 | for why she needed to go home early. | Did Amelia go home late? | 2 |
| 48 | Before little Gloria went to sleep she wanted to hear | a | 89 | an | 0 | story | 87 | excuse | 0 | from her mother. |  |  |
| 49 | At first Victoria did not know why her brother was crying over the sink but then she noticed that he had just sliced | an | 20 | a | 2 | onion | 40 | finger | 50 | with his knife. |  |  |
| 50 | Marie wanted to sample a tiny bit of the sauce so she daintily dipped | a | 27 | an | 0 | finger | 37 | onion | 3 | into the pot. |  |  |
| 51 | After Joanne's first book was published she finally felt she could call herself | an | 75 | a | 25 | author | 90 | success | 3 | in front of her peers. | Was this Joanne's first book? | 1 |
| 52 | Alicia's first client was a failure but her second was | a | 86 | an | 5 | success | 87 | author | 0 | and made her a lot of money. |  |  |
| 53 | Lance had moved to Hollywood in hopes of becoming | an | 70 | a | 25 | actor | 93 | director | 7 | but so far he had only done a few commercials. |  |  |
| 54 | From a young age Steven had an interest in filmmaking and had always dreamed of being | a | 93 | an | 5 | director | 77 | actor | 30 | when he grew up. | Was Steven interested in art? | 2 |
| 55 | Dana enjoyed singing in private but was scared to death at the thought of performing in front of | an | 68 | a | 25 | audience | 93 | crowd | 60 | at the concert. |  |  |
| 56 | Carrie's father had always joked that two is company but three is | a | 100 | an | 0 | crowd | 93 | audience | 0 | for him. |  |  |
| 57 | Damon preferred climbing stairs over going up | an | 34 | a | 11 | escalator | 69 | wheelchair | 0 | because it had given him the chance to get a little exercise. | Did Damon prefer stairs over lifts? | 1 |
| 58 | The doctors told Monica not to use her legs too much so soon after her surgery so she often used | a | 66 | an | 0 | wheelchair | 73 | escalator | 0 | when moving around to rest her legs. | Could Monica use stairs very often? | 2 |
| 59 | The pilot had to make an emergency landing in the middle of the desert because he was nowhere near | an | 52 | a | 16 | airport | 73 | city | 7 | or a safe place to land. |  |  |
| 60 | Hannah wanted to live in a small town but her husband preferred to live closer to | a | 16 | an | 0 | city | 90 | airport | 27 | because of his job. |  |  |
| 61 | Although the basketball team's defense was very strong they did not have so much of | an | 70 | a | 18 | offense | 34 | coach | 0 | since the middle of the season. |  |  |
| 62 | Ever since Mr.Barnes had moved away Bobby's football team had been left without | a | 98 | an | 0 | coach | 70 | offense | 0 | and thus lost all their games. |  |  |
| 63 | The group had been brainstorming all day but they still didn't have | an | 75 | a | 25 | idea | 83 | name | 0 | for their project. | Did the group reach a conclusion? | 2 |
| 64 | The guys didn't know what to call their band so Trent told everyone to come up with | a | 68 | an | 11 | name | 50 | idea | 77 | and they would vote. | Did Trent suggest to vote? | 1 |
| 65 | Being from the mainland Karen never got used to the feeling of living on | an | 73 | a | 14 | island | 93 | ship | 7 | and being so removed from everything. | Was Karen from the mainland? | 1 |
| 66 | Sandra decided that she would work for a cruise line for a year before starting university although she had never been on | a | 100 | an | 0 | ship | 23 | island | 7 | and did not know how to swim. |  |  |
| 67 | As Elizabeth climbed the ladder in the barn she heard a hooting sound coming from the rafters and looked up to see | an | 86 | a | 14 | owl | 87 | cat | 0 | staring down at her. |  |  |
| 68 | Every time they went for walks Sylvia's dog Rex would break into a run as soon as he spotted | a | 82 | an | 18 | cat | 27 | owl | 10 | but luckily Rex couldn't climb trees. |  |  |
| 69 | The first time Timmy saw the Pacific he thought it was a lake but his brother laughed and explained to him that it was | an | 73 | a | 5 | ocean | 93 | mirage | 0 | and he should be careful of the waves. |  |  |
| 70 | As the men stumbled across the desert they thought they saw an oasis on the horizon but it turned out to be | a | 68 | an | 16 | mirage | 57 | ocean | 3 | so they continued walking. | Was there an oasis on the horizon? | 2 |
| 71 | The waitress at the next table was trying to scribble everything down but it was obvious that she did not know how to take | an | 57 | a | 0 | order | 90 | compliment | 0 | from her customers. |  |  |
| 72 | Marcy did not deal well with praise and had never really learned how to take | a | 73 | an | 0 | compliment | 90 | order | 0 | even from her friends. |  |  |
| 73 | Carly wasn't sure if the Venus flytrap was classified as a plant or as | an | 77 | a | 18 | animal | 80 | flower | 7 | but it seemed to have characteristics of both. |  |  |
| 74 | While Natasha was strolling through the colourful gardens she reached down and picked | a | 80 | an | 0 | flower | 93 | animal | 0 | up off the ground. | Was Natasha in the gardens? | 1 |
| 75 | The chemistry teacher explained to the class that water and salt were both compounds but that nitrogen was just | an | 68 | a | 30 | element | 60 | liquid | 0 | that hadn't been combined. |  |  |
| 76 | Orlando thought that glass was a solid but when he held it under the Bunsen burner it melted and turned into | a | 73 | an | 0 | liquid | 90 | element | 0 | that could be sculpted. |  |  |
| 77 | Violet was just learning how to make an omelette so her father began by showing her how to crack | an | 70 | a | 0 | egg | 100 | window | 0 | in the kitchen. |  |  |
| 78 | Because they were playing baseball so close to the house the children ended up shattering | a | 75 | an | 0 | window | 97 | egg | 0 | and destroying a nest that some birds had built on the sill. |  |  |
| 79 | Bob claims to be from outer space but nobody believes that he is actually | an | 95 | a | 0 | alien | 97 | tourist | 0 | from another planet. | Does Bob claim to be from Canada? | 2 |
| 80 | With his khaki shorts and his loud Hawaiian shirt and the camera around his neck you could definitely tell that the man was | a | 95 | an | 5 | tourist | 97 | alien | 0 | in the country |  |  |

| *Supplementary Table 2.* List of changes to the materials used by Delong et al. (2005) | | |
| --- | --- | --- |
| Type of change | Item number | Change |
| Spelling | 5-6  7-8 | ‘check’ > ‘cheque’  ‘airplane’ > ‘aeroplane’ |
| Word replacement | 14  21-22  22  29-30  34 | ‘College’ > ‘university’  ‘Dollar’ > ‘pound’  ‘99-cent store’ > ‘Poundland’  ‘Studio’ > ‘flat’  ‘bike’ > ‘bicycle’ |
| Phrase/Sentence change (underlined) | 57  58 | Old: riding in a/an elevator/wheelchair  New: going up a/an escalator/wheelchair  Old: The doctors would not allow Monica to walk too soon after her surgery so the only way for her to move from place to place was in a/an wheelchair/elevator accompanied by a nurse.  New: The doctors told Monica not to use her legs too much so soon after her surgery she often used a/an wheelchair/escalator when moving around to rest her legs. |
| Sentence-final words | 72 | Original materials did not contain words after the critical noun. We added even from her friends. |
| Comprehension questions |  | We created new comprehension questions as the originals are not publicly available and were not made available upon request. A comparison of response accuracy is therefore uninformative. In addition, an analysis of the obtained responses suggested that several questions were somewhat ambiguous (in particular, items 36, 43, 57), such that what we labeled as an ‘incorrect’ response occurred much more frequently than for other questions, and was so consistently across labs. Subject-level accuracy scores taking into account the ambiguity in these items are available on our OSF webpage (https://osf.io/eyzaq/). |

| *Supplementary Table 3.* Detailed information about participants, trial numbers and EEG recording equipment per laboratory. | | | | | | | | | | | | | |
| --- | --- | --- | --- | --- | --- | --- | --- | --- | --- | --- | --- | --- | --- |
|  | **Tested participants** | | | | | **Analyzed participants** | | | **EEG recording details** | | | | |
| **Laboratory** | **N** | **N Females** | **Mean age (sd) in years** | **N familial left-handedness** | **Question Accuracy as % correct (range)** | **N** | **Mean N article trials** | **Mean N noun trials** | **EEG system** | **Channels (EOG)** | **Recording Reference** | **Online filter (Hz)** | **Sampling rate (Hz)** |
| University of Birmingham | 43 | 35 | 19.2 (2.4) | 4 | 94 (80-100) | 42 | 78 | 78 | ANT Neuro | 64 (1) | CPz | 0.01-150 | 500 |
| University of Bristol | 39 | 24 | 22.8 (4.4) | 12 | 92 (81-100) | 39 | 77 | 77 | Brain Vision Acticap | 32 (0) | FCz | DC-1000 | 1000 |
| University of Edinburgh | 40 | 33 | 21.4 (2.8) | 7 | 94 (80-100) | 37 | 76 | 76 | Biosemi Active-Two | 64 (4) | CMS | DC-100 | 512 |
| University of Glasgow | 40 | 22 | 23.0 (3.2) | 13 | 91 (71-100) | 40 | 79 | 79 | Biosemi Active-Two | 128 (4) | CMS | DC-100 | 512 |
| University of Kent | 38 | 26 | 20.8 (3.1) | 9 | 92 (75-100) | 38 | 78 | 78 | Brain Vision Acticap | 64 (2) | FCz | DC-140 | 1000 |
| University College London | 38 | 22 | 23.4 (3.7) | 10 | 88 (55-100) | 34 | 77 | 77 | Biosemi Active-Two | 32 (2) | CMS | DC-100 | 512 |
| University of Oxford | 37 | 12 | 24.3 (6.3) | 13 | 91 (71-100) | 32 | 76 | 76 | Biosemi Active-Two | 64 (4) | CMS | DC-100 | 1024 |
| University of Stirling | 40 | 20 | 25.6 (4.7) | 13 | 89 (60-100) | 33 | 78 | 78 | NeuroScan SynAmps2 | 64 (4) | CPz | DC | 250 |
| University of York | 41 | 28 | 20.8 (2.1) | 8 | 89 (76-100) | 39 | 76 | 76 | ANT Neuro | 64 (4) | M1 | DC | 500 |
