# Supplementary material for: Large-scale replication study reveals a limit on probabilistic prediction in language comprehension
Source: eLife. 2018 Apr 3;7:e33468. doi: 10.7554/eLife.33468 (PMC5896878; doi:10.7554/eLife.33468)
Supplement: Transparent reporting form [file elife-33468-transrepform.docx]

***eLife’s* transparent reporting form**

We encourage authors to provide detailed information *within their submission* to facilitate the interpretation and replication of experiments. Authors can upload supporting documentation to indicate the use of appropriate reporting guidelines for health-related research (see [EQUATOR Network](http://www.equator-network.org/%20)), life science research (see the [BioSharing Information Resource](https://biosharing.org/)), or the [ARRIVE guidelines](http://www.plosbiology.org/article/info:doi/10.1371/journal.pbio.1000412) for reporting work involving animal research. Where applicable, authors should refer to any relevant reporting standards documents in this form.

If you have any questions, please consult our Journal Policies and/or contact us: [editorial@elifesciences.org](mailto:editorial@elifesciences.org).

**Sample-size estimation**

- You should state whether an appropriate sample size was computed when the study was being designed
- You should state the statistical method of sample size computation and any required assumptions
- If no explicit power analysis was used, you should describe how you decided what sample (replicate) size (number) to use

Please outline where this information can be found within the submission (e.g., sections or figure legends), or explain why this information doesn’t apply to your submission:

- We have an appropriate sample size. We did not perform a sample size computation, because our study is a direct replication study with a sample size (N = 334) that is more than 10 times that of the original N=32 study (we mention this in the Abstract, Introduction page 7 line 10, Methods section on participants and on data processing). Our sample is from 9 different labs, and we aimed to test 40 participants in each lab in order to have at least N=32 in each lab.

**Replicates**

- You should report how often each experiment was performed
- You should include a definition of biological versus technical replication
- The data obtained should be provided and sufficient information should be provided to indicate the number of independent biological and/or technical replicates
- If you encountered any outliers, you should describe how these were handled
- Criteria for exclusion/inclusion of data should be clearly stated
- High-throughput sequence data should be uploaded before submission, with a private link for reviewers provided (these are available from both GEO and ArrayExpress)

Please outline where this information can be found within the submission (e.g., sections or figure legends), or explain why this information doesn’t apply to your submission:

- We performed the same experiment in 9 different laboratories (e.g., methods section on participants)
- ‘biological replication’ does not apply to our submission
- Our criteria for exclusion were pre-registered and are described in the Methods section on data processing

**Statistical reporting**

- Statistical analysis methods should be described and justified
- Raw data should be presented in figures whenever informative to do so (typically when N per group is less than 10)
- For each experiment, you should identify the statistical tests used, exact values of N, definitions of center, methods of multiple test correction, and dispersion and precision measures (e.g., mean, median, SD, SEM, confidence intervals; and, for the major substantive results, a measure of effect size (e.g., Pearson's r, Cohen's d)
- Report exact p-values wherever possible alongside the summary statistics and 95% confidence intervals. These should be reported for all key questions and not only when the p-value is less than 0.05.

Please outline where this information can be found within the submission (e.g., sections or figure legends), or explain why this information doesn’t apply to your submission:

- Our most important statistical analyses were pre-registered, and some additional ones were exploratory. We describe and justify them in the Methods section (Pre-registered Replication analysis, Pre-registered Single-trial analysis, Exploratory Single-trial analyses, and Exploratory Bayesian analyses)
- All the exact values for the Replication analysis are described in the associated Results and Method section, likewise for the single-trial analyses
- All the exploratory analyses use the exact same data and pooling as the Replication analysis or the Single-trial analysis.
- We report exact p-values for each test, except for the correlation analysis in Figure 1. These are 9 (labs) * 22 (electrode channels) * 2 (word type) = 396, which are comprehensively summarized as scalp maps, but the exact values are available on our OSF page, which is linked in the figure legend. Figure 2 does report specific r-values for the analysis with data pooled from all labs.

(For large datasets, or papers with a very large number of statistical tests, you may upload a single table file with tests, Ns, etc., with reference to sections in the manuscript.)

**Group allocation**

- Indicate how samples were allocated into experimental groups (in the case of clinical studies, please specify allocation to treatment method); if randomization was used, please also state if restricted randomization was applied
- Indicate if masking was used during group allocation, data collection and/or data analysis

Please outline where this information can be found within the submission (e.g., sections or figure legends), or explain why this information doesn’t apply to your submission:

In each lab, participants were allocated to condition before they entered the lab. We used a pseudo-randomization such that we would have an equal number of participants assigned to the two versions of the stimulus lists

**Additional data files (“source data”)**

- We encourage you to upload relevant additional data files, such as numerical data that are represented as a graph in a figure, or as a summary table
- Where provided, these should be in the most useful format, and they can be uploaded as “Source data” files linked to a main figure or table
- Include model definition files including the full list of parameters used
- Include code used for data analysis (e.g., R, MatLab)
- Avoid stating that data files are “available upon request”

Please indicate the figures or tables for which source data files have been provided:

Our results and Figures 1, 2, 4,5 and supplementary figures can be reproduced using

data files and scripts that are publicly available on the Open Science Framework

<https://osf.io/eyzaq>.
